# Composition, Succession, and Source Tracking of Microbial Communities throughout the Traditional Production of a Farmstead Cheese

**DOI:** 10.1128/mSystems.00830-21

**Published:** 2021-09-28

**Authors:** Lang Sun, Dennis J. D’Amico

**Affiliations:** a Department of Animal Science, University of Connecticut, Storrs, Connecticut, USA; Teagasc Food Research Centre

**Keywords:** cheese, farmstead, microbiome, milk, source tracking, wood

## Abstract

Prior to the advent of milk pasteurization and the use of defined-strain starter cultures, the production and ripening of cheese relied on the introduction and growth of adventitious microbes from the environment. This study characterized microbial community structures throughout a traditional farmstead cheese production continuum and evaluated the role of the environment in microbial transfer. In total, 118 samples (e.g., raw milk, cheese, and environmental surfaces) were collected from milk harvesting through cheese ripening. Microbial communities were characterized based on amplicon sequencing of bacterial 16S rRNA and fungal internal transcribed spacer genes using the Illumina MiSeq platform. Results indicated that the environment in each processing room harbored unique microbial ecosystems and consistently contributed microbes to milk, curd, and cheese. The diverse microbial composition of milk was initially attributed to milker hands and cow teats and then changed substantially following overnight ripening in a wooden vat to one dominated by lactic acid bacteria, including Lactococcus lactis, *Lactobacillus*, and *Leuconostoc*, as well as fungi such as *Exophiala*, *Kluyveromyces*, and *Candida*. Additional microbial contributions were attributed to processing tools, but the composition of the cheese paste remained relatively stable over 60 days of ripening. In contrast, rind communities that were largely influenced by direct contact with bamboo aging mats showed a distinct succession pattern compared to the interior sections. Overall, these findings highlight the critical role of traditional tools and practices in shaping the microbial composition of cheese and broaden our understanding of processing environments as important sources of microbes in food.

**IMPORTANCE** Throughout the 20th century, especially in the United States, sanitation practices, pasteurization of milk, and the use of commercial defined-strain starter cultures have enhanced the safety and consistency of cheese. However, these practices can reduce cheese microbial diversity. The rapid growth of the artisanal cheese industry in the United States has renewed interest in recapturing the diversity of dairy products and the microbes involved in their production. Here, we demonstrate the essential role of the environment, including the use of wooden tools and cheesemaking equipment, as sources of dominant microbes that shape the fermentation and ripening processes of a traditional farmstead cheese produced without the addition of starter cultures or direct inoculation of any other bacteria or fungi. These data enrich our understanding of the microbial interactions between products and the environment and identify taxa that contribute to the microbial diversity of cheese and cheese production.

## INTRODUCTION

Modern cheese production practices, including sanitation, heat treatment of milk (e.g., pasteurization), and the use of commercial defined-strain starter cultures in cheese production, have enhanced food safety, consistency, and uniformity but have diminished microbial diversity (1). Prior to the advent of commercial starter cultures, the production and ripening of cheese resulted from the introduction and growth of adventitious microbial populations from the farm and manufacturing environments (e.g., air, animal and human skin, and production tools) (2–4). This high microbial taxonomic diversity combined with particular cheese manufacturing methods contributes to the development of unique sensory characteristics in traditional cheeses (5). Although the majority of cheese produced worldwide is currently made on an industrial scale, renewed interest in artisan cheese, which is often defined as cheese made by hand on a small scale using traditional, time-honored practices and tools, is stimulating the broader dairy industry to recapture the diversity in cheese (6). At the same time, the emergence of high-throughput sequencing (HTS) technologies is transforming the study of food microbiology and providing new insights into the diversity and complex role of microbes in cheese production (7).

Recent advances in HTS have increased interest in characterizing microbial communities in food manufacturing ecosystems. Compared to traditional culture-based methods, HTS provides a less biased picture of microbial communities and has enabled more comprehensive surveys and descriptions of microbial composition, succession, and the relationships between “house” and food product microbiota (8, 9). A number of recent studies have provided valuable insights into the potential microbial contributions from ingredients and production environments to finished food products. In the case of dairy products, the microbiota associated with the animal housing environment (10) and animal teats (11) have been shown to influence the microbial composition of raw milk. Similarly, production environments, including equipment, wooden tools, and ripening shelves, have been shown to act as reservoirs for microbes that participate in the cheesemaking and ripening processes (12–14). Most studies, however, have focused on individual and/or static overviews of microbial communities relevant to cheese production and are limited in addressing microbial transfer.

Because microbes originating from the environment and raw materials can become dominant during cheese production, especially in the absence of the intentional addition of defined cultures, it is important to understand the complex diversity, succession, and transmission of microbes along the farmstead cheesemaking continuum. Bethlehem, a Saint-Nectaire-type cheese produced at the Benedictine Abbey of Regina Laudis, is one of the last remaining farmstead cheeses in the United States that is produced using traditional techniques and tools originally developed in the Auvergne region of France. Bethlehem is produced solely from unpasteurized bovine milk that is collected by hand from cows housed on-site using a wooden vat and wooden tools without the addition of starter cultures or any other bacteria or fungi. Thus, the objectives of this study were to (i) describe the microbial ecosystem of a traditional farmstead cheesemaking environment; (ii) characterize the microbial composition and succession during the production of a traditional raw milk farmstead cheese; and (iii) infer pathways of microbial transfer along the cheesemaking continuum.

## RESULTS AND DISCUSSION

### Samples and sequencing output.

In total, 118 samples were collected from the milking barn, the cheesemaking room, and the ripening cellar at the Benedictine Abbey of Regina Laudis in Bethlehem, CT (see Table S1 in the supplemental material). The cows were milked by hand in a tie-stall barn that was located in the same building as the cheesemaking room, separated by a washroom and a short hallway. The ripening cellar was located in an adjacent building. Of the total samples, 94 and 88 samples were successfully sequenced and passed sequence quality filtering for bacterial and fungal communities, respectively. After preprocessing, 7,048 amplicon sequence variants (ASVs) with a total of 2,808,939 high-quality bacterial 16S rRNA V4 gene sequences and 1,894 ASVs with a total 1,343,231 high-quality fungal internal transcribed spacer (ITS2) gene sequences were retained and used for downstream analysis.

10.1128/mSystems.00830-21.2 TABLE S1 Sample metadata. Download Table S1, DOCX file, 0.10 MB. Copyright © 2021 Sun and D’Amico. 2021 Sun and D’Amico. https://creativecommons.org/licenses/by/4.0/ This content is distributed under the terms of the Creative Commons Attribution 4.0 International license.

### Alpha diversity and beta diversity of microbial populations from environmental samples.

**FIG 1:**
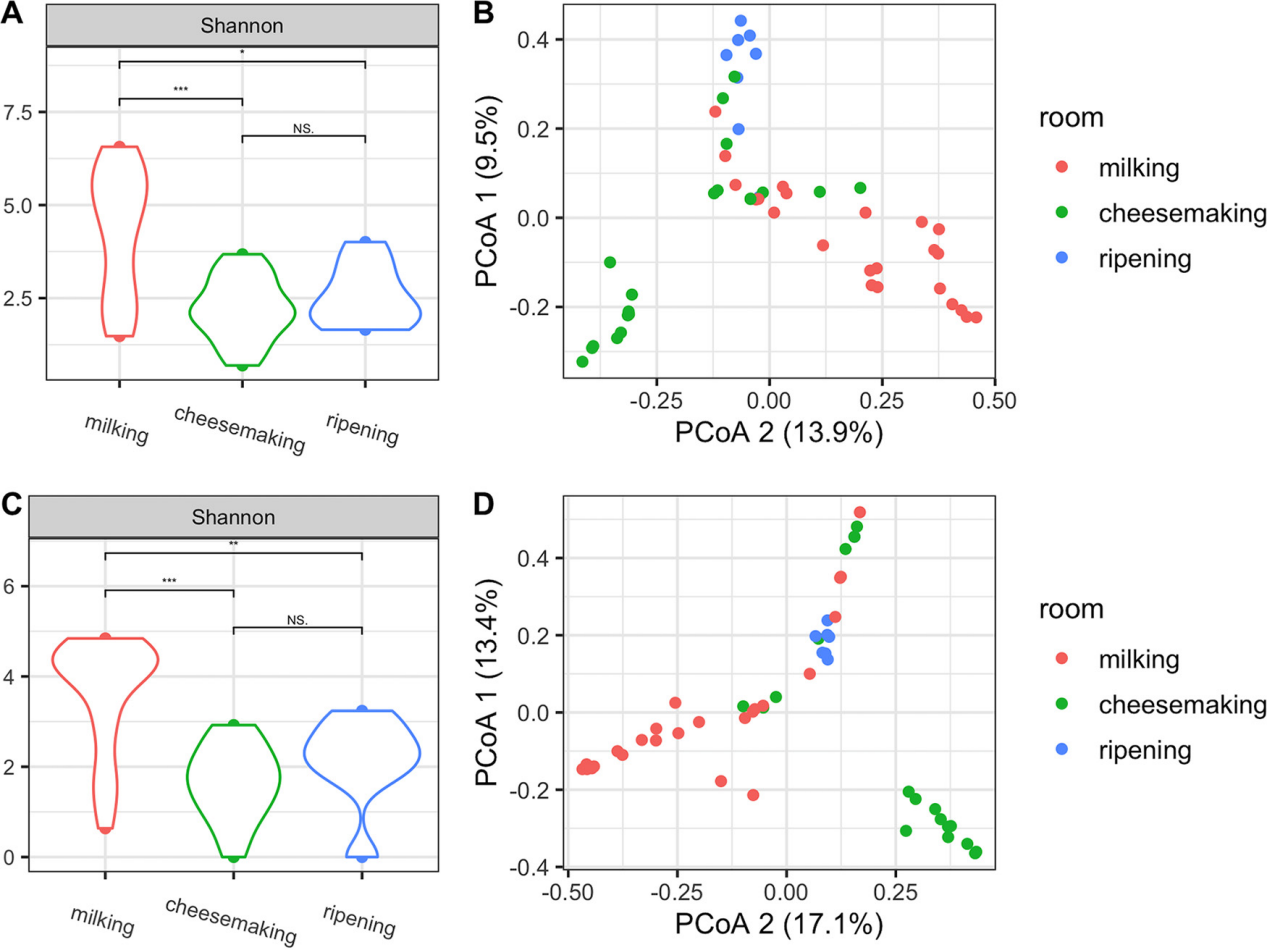
Alpha diversity distributions of bacterial (A) and fungal (C) communities in environmental samples from the three processing rooms along the farmstead cheesemaking continuum. Levels of significance are indicated by asterisks: *, *P < *0.05; **, *P < *0.01; ***, *P < *0.001. Principal coordinate analysis based on Bray-Curtis dissimilarity of bacterial (B) and fungal (D) communities in environmental samples.

Alpha diversity of both bacterial and fungal communities in environmental samples differed between processing rooms, with significantly higher diversity in the milking barn (Kruskal-Wallis, Shannon index; bacteria, df = 2, *P = *5.57e−04; fungi, df = 2, *P = *4.06e−05) (Fig. 1A and C), which could be attributed to the presence of livestock, feed, bedding, and feces that can harbor and disperse highly diverse microbial populations (5). Routine cleaning of sites from which samples were collected in the cheesemaking room and the ripening cellar presumably resulted in lower microbial loads and the lower microbial diversity observed compared to those from the milking barn. The spatial clustering of house microbiota by processing room (Fig. 1B and D) suggests separate and unique microbial ecosystems were established in each room, which was further confirmed by pairwise permutational analysis of variance (PERMANOVA) (Table S2). This is likely due to the differences in types of surfaces sampled and the differences in food production practices in each room (8), as the milking barn was used for milk collection and the cheesemaking room and the ripening cellar were used for cheese production and ripening, respectively.

10.1128/mSystems.00830-21.3 TABLE S2 Processing location effect on microbial beta diversity using pairwise PERMANOVA. Download Table S2, DOCX file, 0.03 MB. Copyright © 2021 Sun and D’Amico. 2021 Sun and D’Amico. https://creativecommons.org/licenses/by/4.0/ This content is distributed under the terms of the Creative Commons Attribution 4.0 International license.

### Microbial composition of environmental samples.

**FIG 2:**
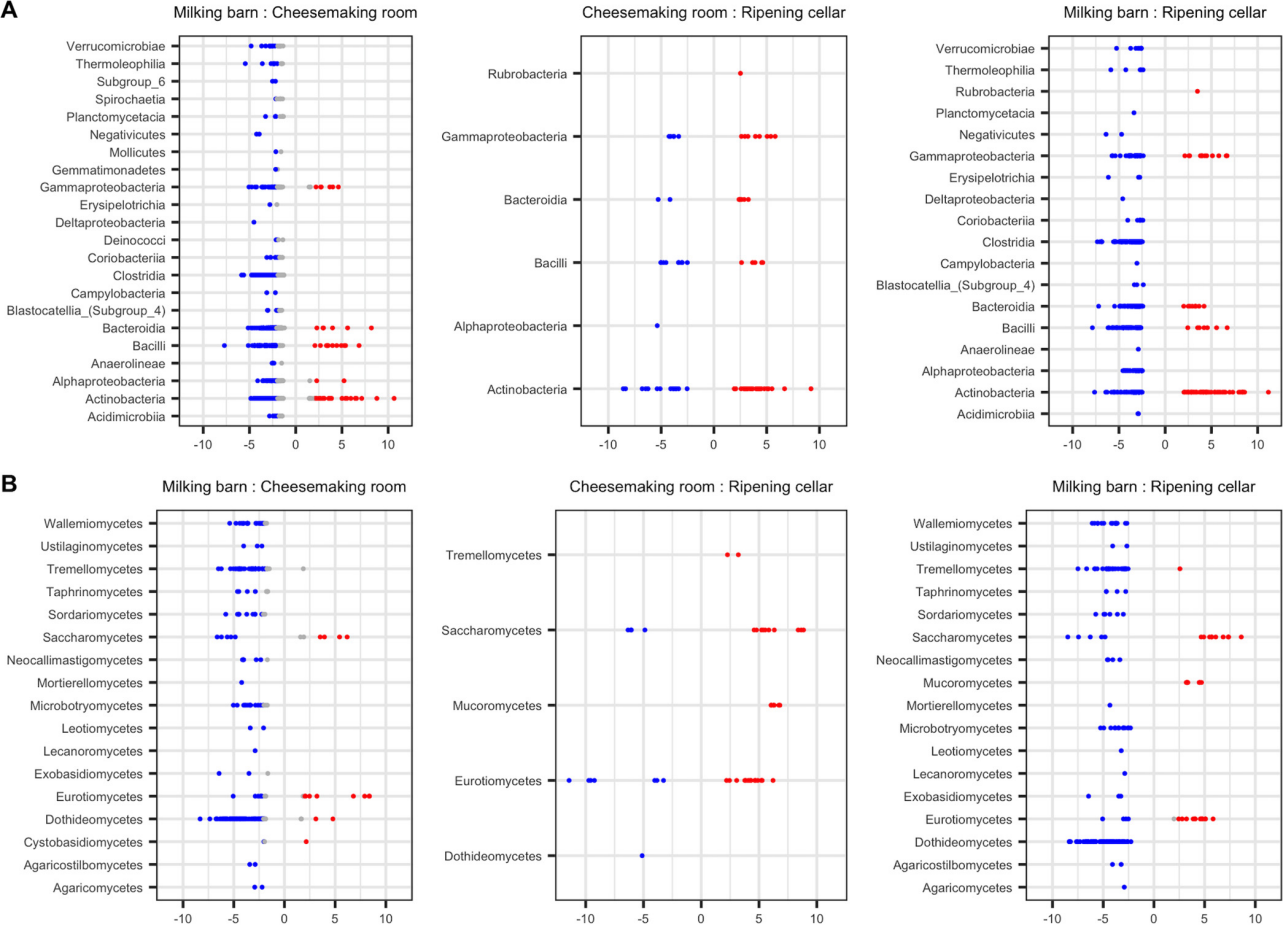
Differential abundance of bacterial (A) and fungal (B) ASVs between processing locations from DESeq2 analysis. Each point represents an ASV that differed between the two environments at an adjusted *P* value of  <0.01. Points are grouped at the class level and are colored as red or blue if they have an absolute log_2_ fold change of >2 for the comparison.

**FIG 3:**
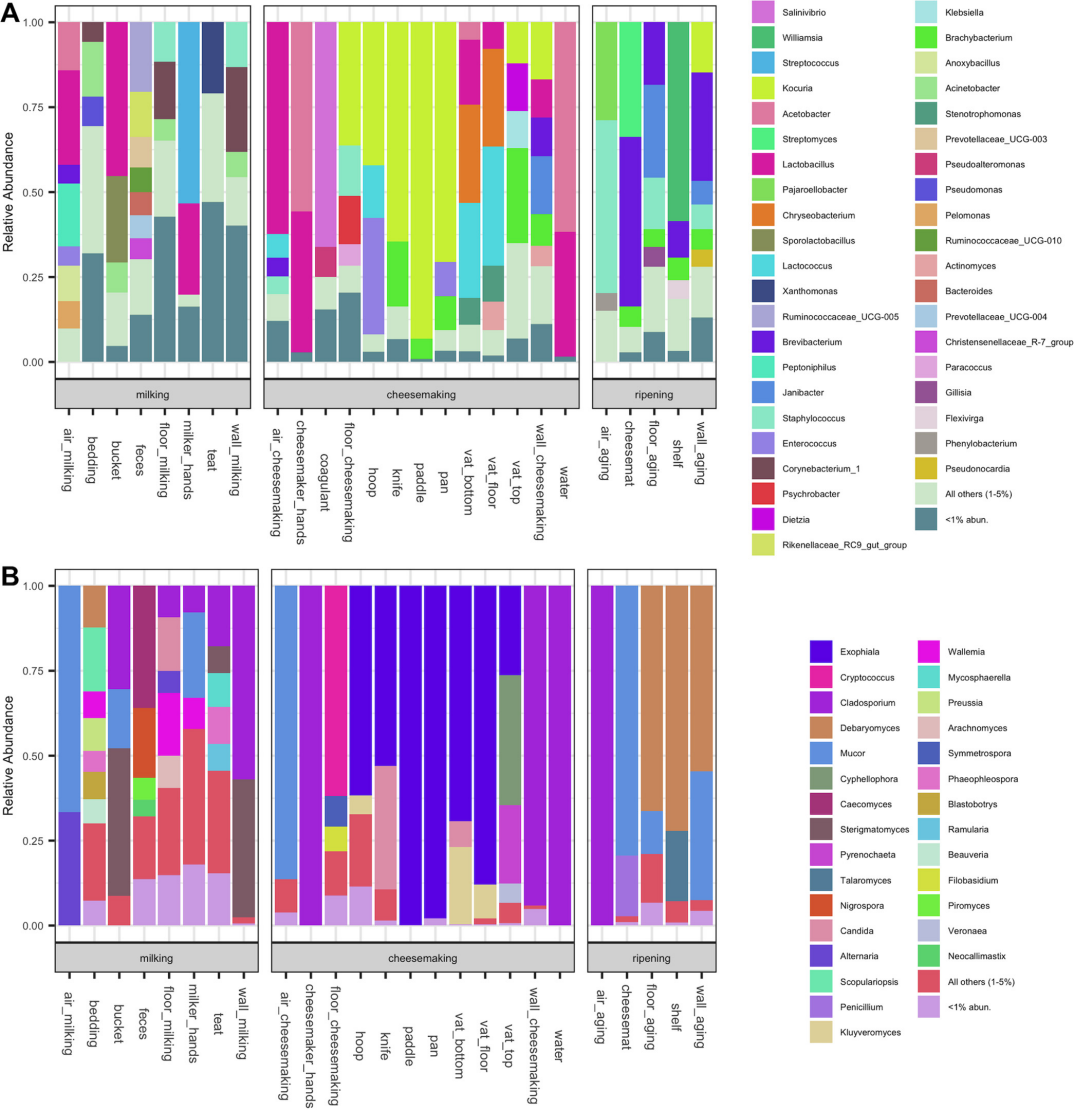
Microbial composition of environmental samples collected from three processing rooms along the farmstead cheesemaking continuum. Vertical columns indicate the average relative abundance of sequences corresponding to bacteria (A) and fungi (B) at the genus level.

Differential abundance analysis was conducted to identify the microbial taxa that were important in forming the unique microbial ecosystems in each processing room. The largest differences in ASV relative abundance were observed between the milking barn and the other two rooms (Fig. 2). In total, 365 bacterial ASVs belonging to 17 classes and 159 fungal ASVs belonging to 16 classes were more abundant in the milking barn than the other two rooms. The majority of the bacterial ASVs were anaerobic gut-associated taxa, with 32% belonging to class *Clostridia* (e.g., *Ruminococcaceae* and *Christensenellaceae*) and 18% to *Bacteroidia* (e.g., *Prevotellaceae*, *Bacteroides*, and *Rikenellaceae*). These taxa were prevalent across environmental samples in the milking barn, with the highest relative abundance in feces (Fig. 3A). Another 25% of the differentially abundant bacterial ASVs were *Gammaproteobacteria* (e.g., Acinetobacter) and *Actinobacteria* (e.g., *Corynebacterium* and *Arthrobacter*). These taxa were previously found on cow teats or teat apices (4, 15). Differentially abundant bacterial ASVs belonging to *Bacilli* (e.g., *Aerococcus* and *Jeotgalicoccus*) and fungal ASVs belonging to *Dothideomycetes* (e.g., *Cladosporium* and *Mycosphaerella*) were previously reported as abundant in the air or airborne dust on a cattle breeding farm (16). However, in the present study, these fungal taxa were abundant in the bedding, floor, walls, and cow teats but not in the air, which was dominated by *Mucor* and *Alternaria* (Fig. 3B). Overall, the prevalence of the differentially abundant taxa in the milking barn is likely attributed to the presence of cows and the open farm environment. Coagulase-negative Staphylococcus was also identified across several environmental samples. Notably, Staphylococcus equorum, which is commonly identified in cheese rinds (17), was abundant in the milking barn (e.g., wall, floor, teats, etc.) (18).

Twenty-six bacterial ASVs and 11 fungal ASVs were differentially more abundant in the cheesemaking room than the other two processing rooms, including bacterial taxa that are technologically important to cheese production (e.g., Lactococcus lactis, Enterococcus italicus, and *Lactobacillus* spp.). These taxa were widely distributed in the cheesemaking room but were especially dominant in specific samples (e.g., Lactococcus lactis in the wood vat, cheese hoop, and air samples; Enterococcus italicus on the metal pan and cheese hoop; and *Lactobacillus* spp. in samples from the wood vat, cheesemaker hands, water, and the wall of the cheesemaking room) (Fig. 3A). ASVs belonging to *Actinobacteria* were also represented in the cheesemaking room. Notably, *Kocuria* spp. were dominant across the cheesemaking tools except for the lower sections of the wooden vat. Kocuria rhizophila, Kocuria carniphila, Kocuria varians, Kocuria kristinae, and undefined *Kocuria* species have been isolated from surface-ripened cheeses, including Saint-Nectaire, Reblochon, and Tilsit (19, 20), but their technological roles are not clear. *Kocuria* spp. were also previously identified in wooden vats, including Kocuria rhizophila in Gerles used for Salers production in France (21) and *Kocuria kristiniae* in Tinas used for the production of Ragusano in Sicily (12). Although the microbial composition of the wooden vat was similar to that of previous studies (12, 21), variation in microbial composition between sections was additionally observed here. The top section of the vat was dominated by *Brachybacterium* spp., *Dietzia* spp., *Kocuria* spp., and Klebsiella sp., whereas the other two sections were more similar and were dominated by *Lactobacillus* spp., Lactococcus lactis, *Chryseobacterium* spp., and *Stenotrophomonas* spp. These differences are likely a function of the bidirectional interaction between the wood and the liquid milk and whey (2, 21, 22), whereby frequent contact with, and absorption of, milk and whey could selectively enrich for specific taxa.

*Exophiala* (class *Eurotiomycetes*) and *Kluyveromyces* (class *Saccharomycetes*) were differentially abundant fungi in the cheesemaking room. *Kluyveromyces* are widely identified in the environment, but only Kluyveromyces lactis and Kluyveromyces marxianus have been found in dairy-associated samples (23). In the present study, Kluyveromyces lactis and Kluyveromyces marxianus were especially dominant in the bottom wall and floor sections of the vat and the cheese hoop. *Exophiala dermatitidis* and *Exophiala phaeomuriformis* have previously been isolated from wood in hot and high-moisture environments, including sauna facilities and steam baths (24, 25), suggesting that its prevalence in the cheesemaking room is associated with the use of wood materials in cheese production.

Forty-four bacterial ASVs and 29 fungal ASVs were identified as more abundant in the ripening cellar than the milking barn and cheesemaking room. The majority of the bacterial ASVs identified belong to the class *Actinobacteria* (Fig. 2A), including *Streptomyces* spp. (dominant on the cheese mat), *Brevibacterium* spp. (dominant in multiple samples), *Pseudonocardia* spp. (dominant on the cheese mat and wall), and *Kocuria* spp. (dominant on the wall) (Fig. 3A). *Streptomyces* was previously identified in cheese, and some strains have demonstrated antifungal effects on cheese (26). *Pseudonocardia* also has demonstrated effects against fungi (27) but is not commonly found in cheese-related environments. *Brevibacterium* is regularly found on cheese rinds, and its abundance in the ripening cellar is likely associated with bidirectional transfer during the cheese-ripening process (8, 13). Differentially abundant ASVs of *Kocuria* in the ripening cellar differed from those that were more abundant in the cheesemaking room, which may be the result of variations within the genus. The differentially abundant fungi in the ripening cellar were *Debaryomyces* (class *Saccharomycetes*), *Penicillium* (class *Eurotiomycetes*), and *Mucor* (class *Mucoromycetes*) (Fig. 2B), which are all important for rind development on certain cheeses, including Bethlehem and Saint-Nectaire (28, 29).

Differential abundance analysis was also conducted using a compositional analysis tool, ALDEx2, that models count data as transformed probability distribution for analysis. Fewer recalls were identified by ALDEx2 (Fig. S1), but the identified ASVs were consistent with DESeq2 results. The only ASV found to be more abundant in the milking barn was the fungus *Paraconiothyrium* (class *Dothideomycetes*), which is widely present in soil environments (30). Its presence in samples from the cow teats, feces, and milking barn floor in the present study may be due to the open environment and the presence of soil in the milking barn. Similar to DESeq2 results, ASVs classified as *Actinobacteria* were more abundant in both the cheesemaking room and the ripening cellar than the milking barn, and taxa associated with cheese ripening were found to be more abundant in the ripening cellar.

10.1128/mSystems.00830-21.1 FIG S1 Differential abundance of bacterial (A) and fungal (B) ASVs between processing locations from ALDEx2 analysis. Each point represents an ASV and is grouped at the genus level. Points are colored red or blue if they showed an effect size of >1 for the comparison. Download FIG S1, EPS file, 0.04 MB. Copyright © 2021 Sun and D’Amico. 2021 Sun and D’Amico. https://creativecommons.org/licenses/by/4.0/ This content is distributed under the terms of the Creative Commons Attribution 4.0 International license.

### Alpha diversity and beta diversity of microbial populations in dairy samples through the farmstead cheesemaking process.

**FIG 4:**
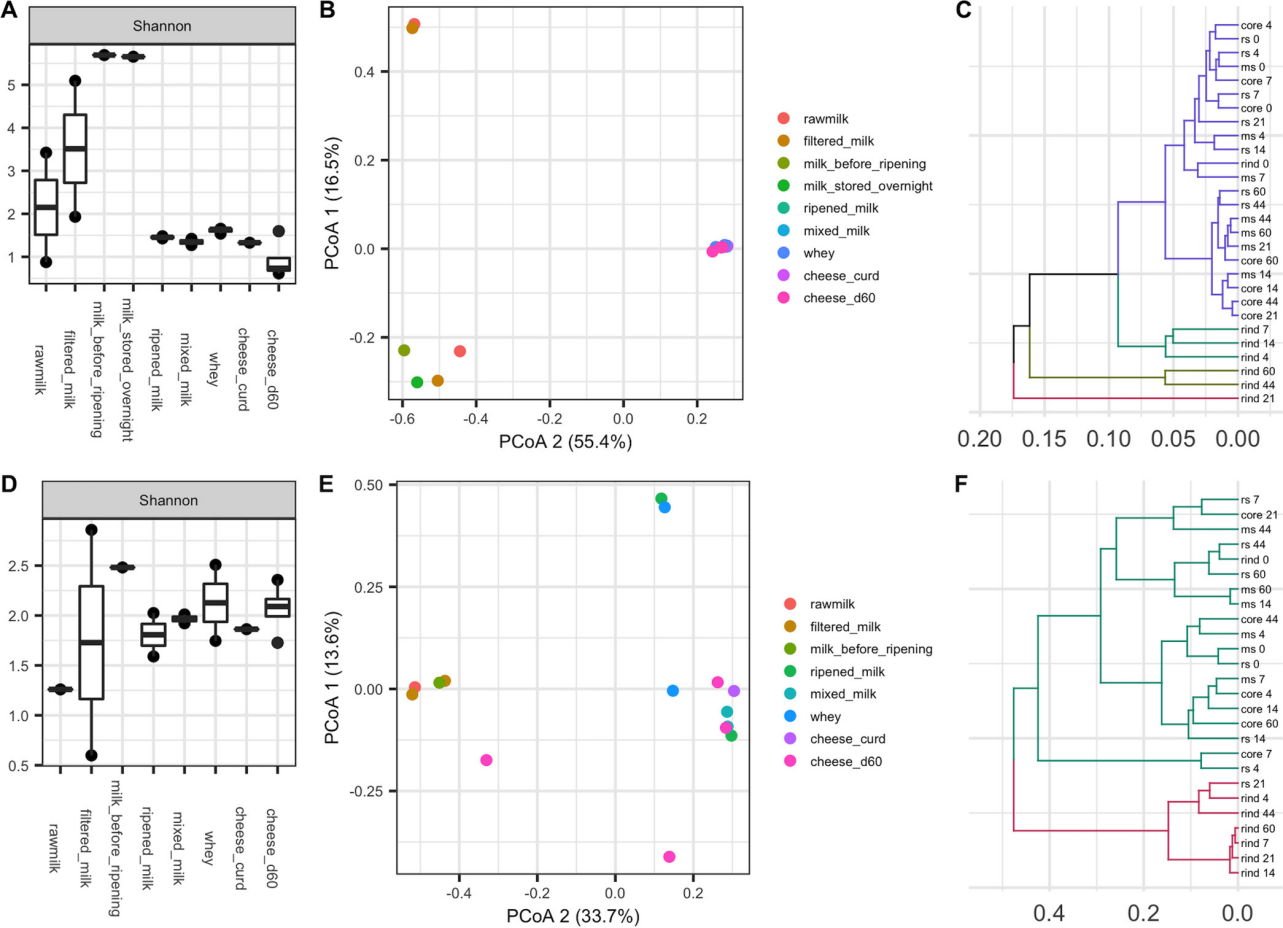
Alpha diversity distributions of bacterial (A) and fungal (D) communities in dairy samples at sequential processing steps along the farmstead cheesemaking continuum. Principal coordinate analysis based on Bray-Curtis dissimilarity of bacterial (B) and fungal (E) communities in dairy samples. UPGMA clustering based on bacterial (C) and fungal (F) weighted UniFrac distances of different cheese sections over time (ms, middle section; rs, rind section).

The alpha diversity of microbial communities in milk increased after the raw milk was commingled and filtered and then decreased following the initial ripening process (“seasoning”) in the wooden vat (Fig. 4A and D). The increase in alpha diversity in filtered milk could be explained by exposure to the highly diverse microbial communities in the milking barn environment (Fig. 1A and C). Clustering dairy samples using principal coordinate analysis (PCoA) revealed a strong microbial shift during the overnight ripening process (Fig. 4B and E). More specifically, ripened milk and samples collected thereafter clustered together and separate from raw milk, filtered milk, milk stored overnight, and milk before ripening. Cluster analysis of cheese samples over 60 days of cheese ripening revealed a temporal development of rind microbial communities (Fig. 4C and F). Bacterial community structures in the rind section gradually became distinct from the interior sections over time, whereas the interior sections remained similar during the ripening process. Four stages of rind development were observed as days 0 to 4, days 4 to 14, days 21 to 44, and days 44 to 60 (Fig. 4C). Fungal communities developed more rapidly on the rind as the rind section was distinct from interior sections as early as day 4 and clustered with the rind thereafter. Like bacteria, the fungal communities in the remaining interior sections clustered together and remained relatively stable over time (Fig. 4F).

### Microbial composition and community succession in dairy samples through the farmstead cheesemaking process.

**FIG 5:**
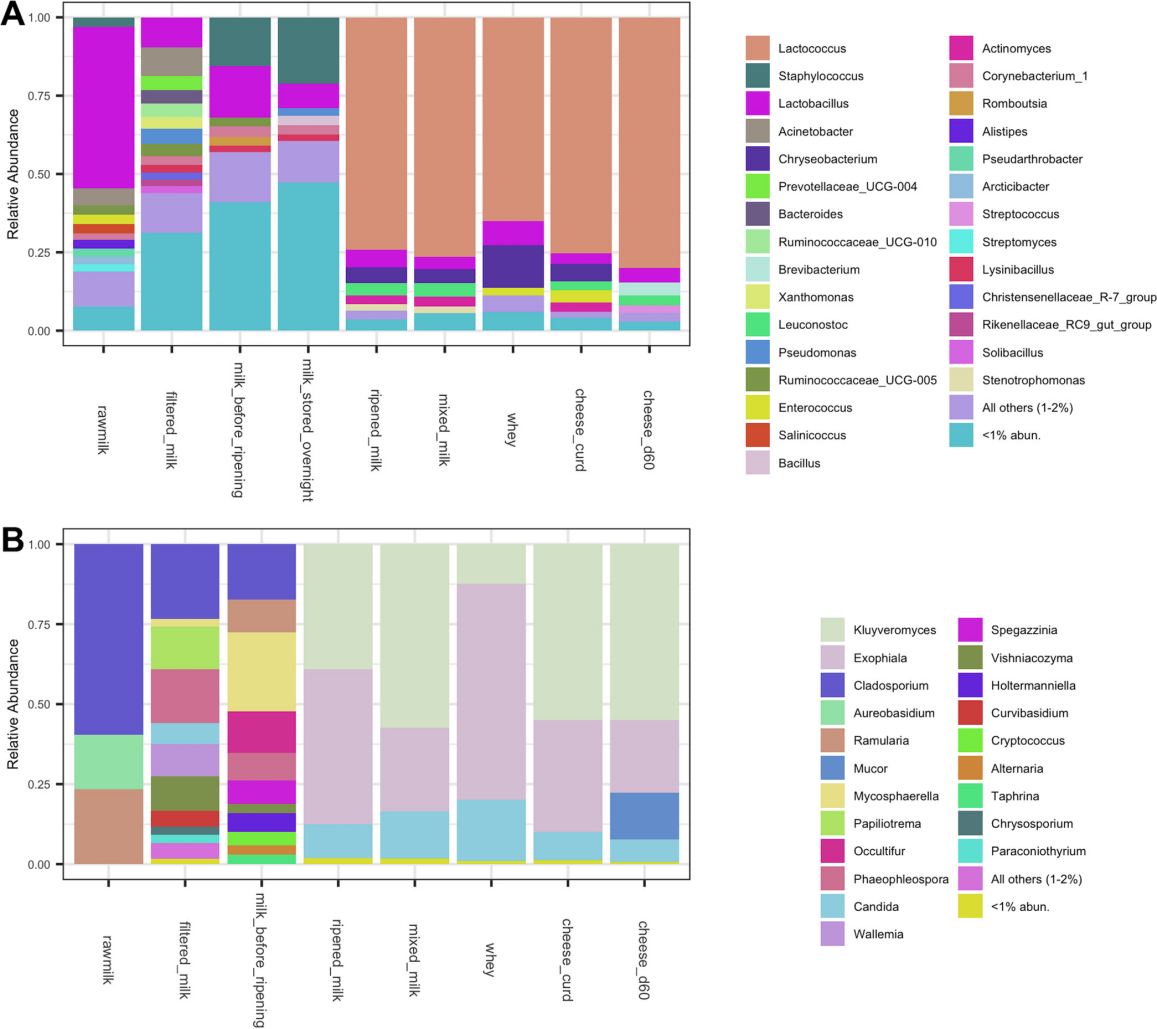
Microbial composition of dairy samples at sequential processing steps along the farmstead cheesemaking continuum. Vertical columns indicate the relative abundance of sequences corresponding to bacteria (A) and fungi (B) at the genus level.

**FIG 6:**
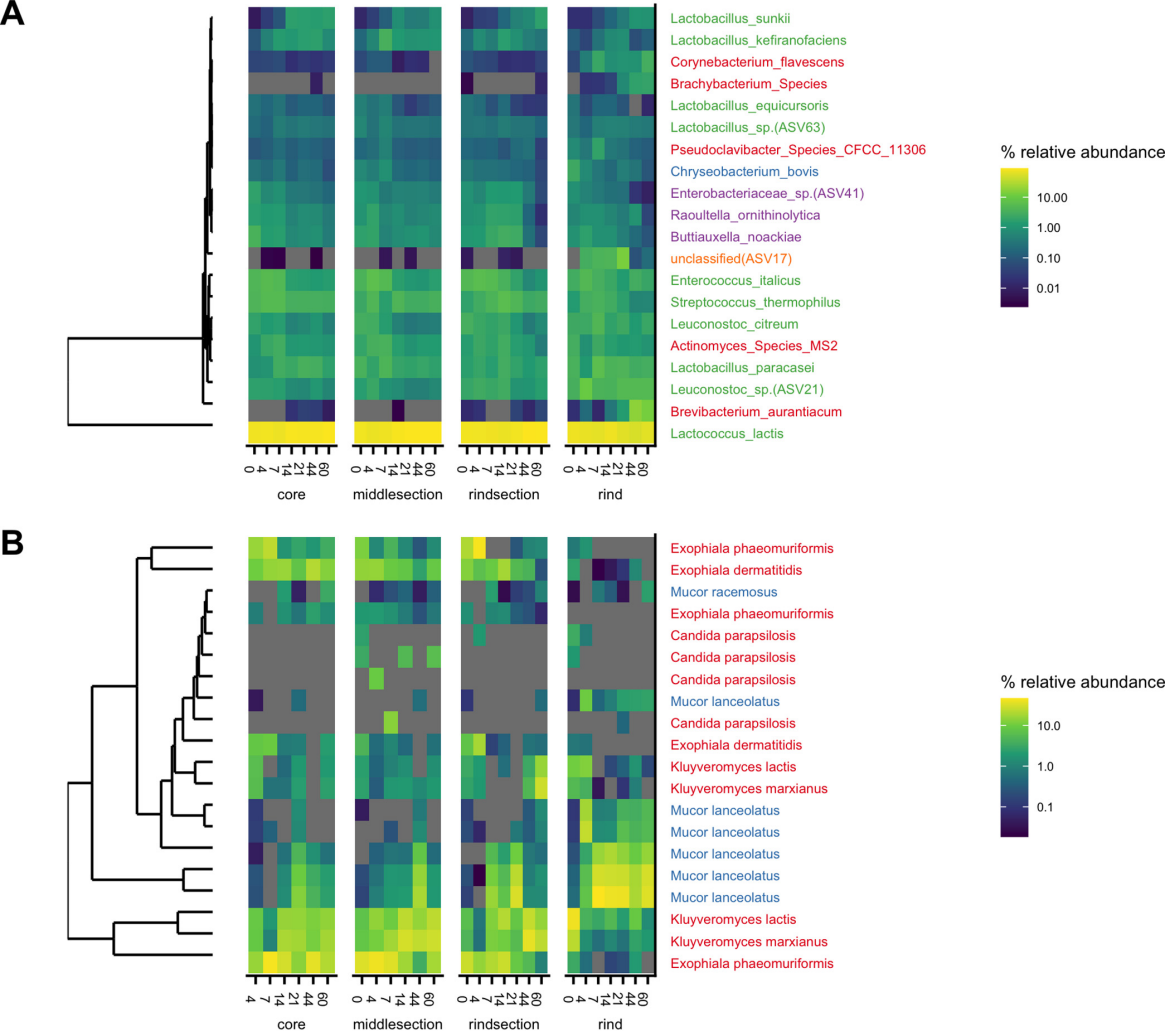
Top 20 bacterial (A) and fungal (B) ASVs in cheese during 60 days of ripening. ASVs were clustered by relative abundance. Species are coded by color at the phylum level (bacteria, red [*Actinobacteria*], green [*Firmicutes*], blue [*Bacteroidetes*], purple [*Proteobacteria*], orange [unknown]; fungi, red [*Ascomycota*], blue [*Mucoromycota*]). Bacterial species assignments were based on the dairy database (DAIRYdb). ASVs not identified in the dairy database were marked with its ASV number.

Selective and differential culture-based enumeration and identification was beyond the scope of the present study, so the taxonomic composition of dairy samples collected at key processing steps are shown as relative abundance based on culture-independent sequencing (Fig. 5). Approximately 50% of the bacteria identified in the raw milk in the present study were *Lactobacillus*, including Lactobacillus paracasei and an unidentified *Lactobacillus* sp. This is not unexpected, considering hygienically collected raw milk is typically comprised of lactic acid bacteria (LAB), streptococci, staphylococci, micrococci, and, to a lesser extent, coryneforms (31). The relative abundance of rare taxa (<1%) increased following subsequent exposure to the environment through collection, filtering, commingling, and refrigerated storage overnight, demonstrating acquisition of microbes along the process. This also helps explain the increase in alpha diversity in dairy samples as previously noted (Fig. 4A and D). A single Staphylococcus ASV identified in the raw milk that also increased in relative abundance was Staphylococcus succinus, which was previously isolated from surface-ripened cheese (32) and traditional fermented sausage (33). Overnight ripening of milk in the wooden vat resulted in a major shift in microbial community composition to one comprised of abundant taxa similar to those from the wooden vat, including Lactococcus lactis, *Lactobacillus* spp., and *Leuconostoc* spp. Overnight fermentation appears to have supported the growth of these taxa, effectively crowding out other low-abundance taxa, resulting in the observed decrease in alpha diversity. A previous study has shown that contact with wood biofilms for 10 min can inoculate milk with additional 4 to 6 log CFU/ml of LAB (2), and that these taxa remained dominant throughout cheesemaking, with LAB populations reaching 9 log CFU/g at the first day of ripening (34, 35). *Enterococcus* was not identified as abundant in the ripened milk, but its population increased during cheesemaking and was eventually abundant in the cheese curd. It was no longer abundant at the genus level in cheese after 60 days of ripening, but Enterococcus italicus was still identified among the top 20 ASVs found in cheese samples (Fig. 6A). *Chryseobacterium* spp. and *Stenotrophomonas* spp. were abundant in samples during cheesemaking but later decreased during cheese ripening. These two genera were also abundant in the core of Saint-Nectaire cheese (36), and their abundance also decreased over time in another uncooked pressed cheese (37). The function of these and other Gram-negative bacteria in cheese production is still not clear. Gram-positive bacteria such as *Brevibacterium* spp. and Streptococcus thermophilus became dominant in the final cheese as the likely result of their salt tolerance and growth of the former on the rind (Fig. 6A). Although Bethlehem is dry salted and the rind is not washed, the presence of *Brevibacterium* spp. is commonly associated with washed rinds (29) and cheese production using brines with high salinity (8). *Brevibacterium* spp. were also widely distributed in the ripening cellar, where environmental conditions may favor their growth and further facilitate their development on the cheese rind.

Fungal populations in raw milk are often present at 2 to 4 log CFU/ml and are typically comprised of technologically important genera, such as *Candida*, *Debaryomyces*, *Geotrichum*, *Kluyveromyces*, *Cladosporium*, *Mucor*, Fusarium, and *Penicillium* (38). Fungal population levels are typically stable during cheesemaking but can increase to 6 to 9 log CFU/g in the final cheese as initially present fungi grow and adventitious fungi are further acquired during cheese ripening (20, 34). Fungal communities in the raw milk in the present study were of low diversity and were comprised mostly of *Cladosporium*, *Aureobasidium*, and *Ramularia* (Fig. 4D and 5B). Fungal diversity increased substantially following increased exposure in the milking barn environment and decreased after overnight ripening to one dominated by a few genera, including *Kluyveromyces*, *Exophiala*, and *Candida*. Kluyveromyces lactis and Kluyveromyces marxianus are commonly identified in dairy samples (23), and Kluyveromyces lactis is often added as a ripening culture in cheese production to metabolize residual lactose in the cheese curd (39). *Candida* is also prevalent in cheeses, with some strains demonstrating strong proteolytic and lipolytic activity (40, 41). *Exophiala* has not been identified in previous analyses of this type of cheese or in wooden vats and is generally associated with spoilage of dairy products (42). In the present study, *Exophiala* spp. were abundant in most of the environmental samples in the cheesemaking room and highly abundant in the whey but gradually decreased in cheese over time. Its role, if any, in Bethlehem cheese is yet to be determined. *Mucor* became dominant in cheese after 60 days of cheese ripening. Although *Mucor* species are often associated with the spoilage of soft cheeses (43) and is undesirable in Saint-Nectaire produced on an industrial scale and/or from pasteurized milk, it is technologically important for the traditional production of uncooked cheeses, including farmstead Saint-Nectaire, Tomme de Savoie, and Taleggio (43, 44), as some *Mucor* species produce lipases and proteases that improve texture and flavor (45, 46). Additional fungal genera identified at low abundance in the final cheese, including *Debaryomyces* (0.26%), *Penicillium* (0.01%), and *Cladosporium* (<0.01%), have been identified in Bethlehem and Saint-Nectaire cheeses (28, 29). However, some differences compared to the previous studies were also noted. Notably, *Geotrichum* was not identified in any sample in the present study, and *Trichothecium* was only identified in the floor of the aging room at a very low level. This may be related to seasonal variations (10, 47), since samples in the present study were only collected in late autumn (time of production) and early winter (ripening cheese).

Observing changes in relative abundance of specific taxa within each cheese section over time (Fig. 6, top 20 ASVs plotted) revealed that Lactococcus lactis maintained >50% relative abundance across all sections throughout cheese ripening. In contrast, some bacteria, including *Brevibacterium auranticum*, a *Brachybacterium* sp., Corynebacterium flavescens, and an unclassified ASV, grew preferentially on the rind. Coryneforms like *Brevibacterium*, *Brachybacterium*, and *Corynebacterium* are important ripening cultures for the production of volatile compounds in cheese (48). They are acid sensitive and salt tolerant and typically grow on cheese rinds following initial fungal growth and the subsequent increase in surface pH (49, 50). We also observed trends in relative abundance over time. For example, five *Lactobacillus* species that were identified among the top 20 ASVs showed differing patterns during ripening (Fig. 6A). Consistent with previous studies, homofermentive *Lactobacillus* (Lactobacillus equicursoris) decreased gradually during ripening, while other “nonstarter” lactobacilli (Lactobacillus sunkii and Lactobacillus paracasei) increased or remained abundant through 60 days (51, 52). Lactobacillus equicursoris is closely related to Lactobacillus delbrueckii and possesses a major cell-bound protease that is responsible for the degradation of casein (53), whereas Lactobacillus sunkii is a recently identified species isolated from a traditional Japanese spontaneously fermented nonsalted pickle product (54). The roles of many diverse *Lactobacillus* species in cheese production are not well known and warrant further investigation. Some fungi developed rapidly on cheese rinds. For example, *Mucor lanceolatus* reached high relative abundance as early as day 4 (28). On the other hand, *Exophiala phaeomuriformis*, *Exophiala dermatitidis*, Kluyveromyces lactis, and Kluyveromyces marxianus were abundant in the interior sections of the cheese, and the relative abundance of *Exophiala* spp. gradually decreased during ripening (Fig. 6B).

### Microbial source attribution.

**FIG 7:**
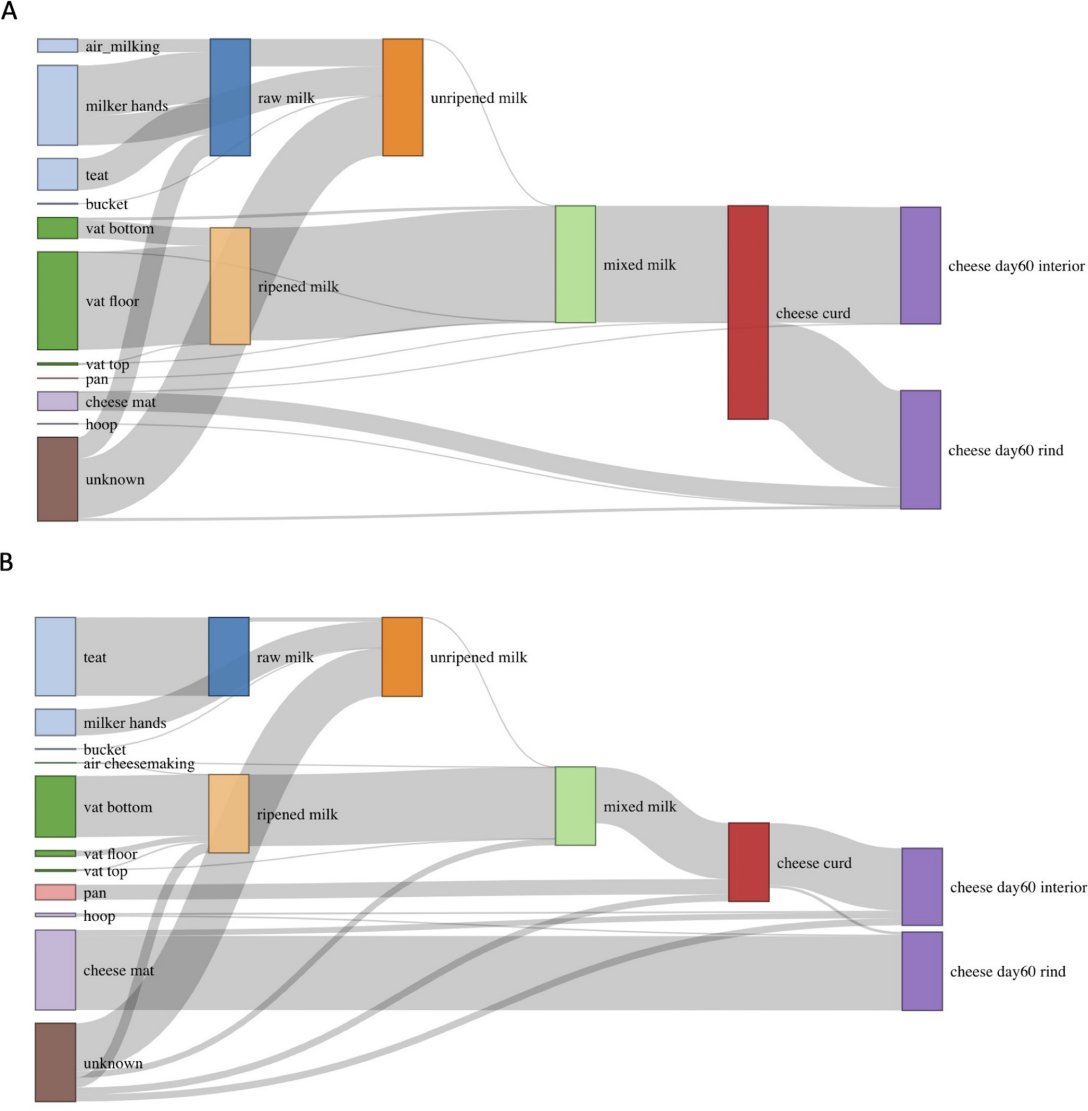
Relative attribution of bacterial (A) and fungal (B) transfer along the farmstead cheesemaking continuum. Environmental source samples are represented on the left, and dairy samples, as sinks and sources, are shown to the right. The height of individual gray flows between bars illustrates the average predicted contribution/proportion of microbes from source samples to the microbial community of respective sink samples. The height of the individual bars of sink samples on the right (dairy samples) sums up to 100%. The height of individual bars of source samples on the left represents the sum of proportions to each of the sink samples.

In order to characterize microbial transmission along the farmstead cheese production continuum, source attribution analyses were conducted in a stepwise order following the cheesemaking process using the SourceTracker tool (55). SourceTracker uses a sampling algorithm to examine the likely distribution of ASVs within user-defined sources, which are then used to determine the affiliation within sample communities (i.e., sinks); taxon distribution is used to determine the attribution from each source. Thus, SourceTracker not only qualitatively identifies possible microbial sources but also quantitatively estimates the proportion of source contributions to a sink. Only environmental samples in direct contact with dairy samples were included in this analysis, such that direct environmental samples as well as the dairy sample from the preceding step were considered potential sources (Fig. 7). SourceTracker attributed 43% of the bacteria in raw milk to the milker’s hands, followed by teats (27%), air in the milking barn (11%), and unknown sources (18%). More specifically, the presence of Lactobacillus helveticus in raw milk was attributed to milker’s hands and air, whereas Acinetobacter lwoffii, Staphylococcus succinus, a *Ruminococcaceae* sp., a *Corynebacterium* sp., and others were attributed to cow teats. Cow teats were estimated to be the only source of fungi in the raw milk, contributing *Cladosporium sphaerospermum*, *Ramularia pratensis*, and *Aureobasidium pullulans*. These source attributions were not unexpected, as environmental contaminants in soil, feces, and bedding material can attach to the exterior of teats and enter milk during collection through milker hands and milking equipment (31). Approximately 23% of the bacteria in the unripened milk (filtered milk, milk before ripening, and milk stored overnight combined) were attributed to carryover from the raw milk, with another 25% introduced from milkers’ hands. The estimated contribution from the milk collection bucket was low (0.7%), and the remaining bacteria were attributed to unknown sources (51%). Unknown sources were also responsible for the largest proportion of fungi in unripened milk (61%), followed by milkers’ hands (34%), raw milk (5%), and the bucket (0.7%). The relatively high contribution from unknown sources could be attributed to sites that were not sampled, including the stainless steel pails used to collect milk during hand milking and the milk filter.

Previous studies have shown that biofilms developed on wooden vat surfaces rapidly inoculate raw milk with high levels of technologically important microbes (2, 14, 21). The data presented here further support this, as our analysis attributed nearly all the bacteria in the ripened milk to the wooden vat (floor [84%], bottom wall [15%], and top wall [0.7%]), with no contribution from the milk originally added to the vat. Both the floor and the bottom wall sections of the vat contributed Lactococcus lactis to the ripened milk. The floor also contributed Chryseobacterium bovis, an *Actinomyces* sp., Lactobacillus paracasei, and Leuconostoc citreum, and the bottom wall was the major source of Acetobacter orientalis and Lactobacillus kefiranofaciens. The limited contribution from the top wall was mostly *Proteobacteria*. Similarly, approximately 87% of fungal communities were attributed to the wooden vat (floor [7%], bottom wall [78%], and top wall [7%]), followed by unknown sources (13%) and the air in the cheesemaking room (0.1%). This included Kluyveromyces marxianus and Kluyveromyces lactis from the bottom wall and *Exophiala dermatitidis* and *Exophiala phaeomuriformis* from both the bottom wall and the floor of the vat. Although the wooden vat played a substantial role in transferring microbes overall, bacteria were mostly derived from the vat floor, whereas fungi were mostly derived from the bottom wall section. The dominant microbial taxa were similar between the two sections, but aerobic microbes were more abundant on the bottom wall than the vat floor, including Acetobacter orientalis, *Kluyveromyces* spp., and Candida parapsilosis. The vat floor was dominated by LAB and *Actinomyces*, which are facultative anaerobic microbes (Fig. 3). The differences between sections may be related to oxygen availability or microbial activity during overnight ripening, but further investigation is needed to confirm this. Interestingly, the vat biofilm in the present study was originally developed through the repeated fermentation of raw unripened milk, yet no contribution from the unripened milk was predicted here. It is likely that the microbial communities in the unripened milk provided a diverse species pool for the biofilm development followed by microbial selection in the mature biofilm reinforced through repeated use of the wooden vat during cheese production (56, 57). Further studies into microbial selection in biofilm development are needed.

In the next step, the ripened milk was mixed with fresh filtered milk and milk stored overnight under refrigeration. Most bacteria in this mixed milk were attributed to the ripened milk (96%) and the vat (3%), whereas the contribution from unripened milk was as low as 0.2%. Fungi in the mixed milk were also predominantly attributed to the ripened milk (91%), followed by unknown sources (8%), unripened milk (0.5%), air (0.1%), and the top section of the vat (0.03%). The limited contribution of microbes from the unripened milk in both cases is likely due to the differences in microbial biomass between the ripened milk and unripened milk, whereby the influence of samples with relatively lower microbial loads is limited despite the disproportionately higher volume. After addition of coagulant, hand breaking of curds, removal of whey, and addition of water, more than 99% of the bacteria in the resulting cheese curd were attributed to those carried over from the mixed milk, with the remaining 0.3% (including Kocuria kristinae and Enterococcus italicus) attributed to the metal pan used during whey removal and pressing of the curd mass under the whey. The contribution of *Enterococcus* from the metal pan is likely responsible for the observed increase in the relative abundance of *Enterococcus* after cheesemaking (Fig. 5A). Similar observations were made for fungal communities in the cheese curd, with major contributions from mixed milk (72%) and unknown sources (9%) and a relatively higher contribution from the metal pan (19%; mostly *Exophiala dermatitidis* and *Exophiala phaeomuriformis*).

Analysis of the bacterial contributions to the finished cheese after 60 days of ripening was separated by cheese interior and rind sections. The bacterial composition of the interior cheese paste was mostly attributed to the cheese curd (99%) with a minor contribution from the cheese mat (0.03%). The majority contribution to the rind was also attributed to the cheese curd (83%), with higher contributions from the cheese mat (16%) and additional contributions from the cheese hoop (0.2%; Enterococcus italicus, Kocuria kristinae, and Lactococcus lactis) and unknown sources (2%). The cheese mat was also identified as the major source of Brevibacterium aurantiacum, Brevibacterium casei, and a *Brachybacterium* sp. on the cheese rind over the course of cheese ripening. Fungal contributions were similar to those observed for bacteria, whereby the majority of fungi in the cheese paste were contributed by the cheese curd (79%), followed by unknown sources (11%), the cheese mat (7%), and the hoop (2%). In contrast, fungi on the cheese rind were attributed to the cheese mat (95%) with limited contribution from the cheese curd (4%) and the hoop (1%). The presence of *Mucor lanceolatus*, and the subsequent high relative abundance on the cheese rind, was linked to the cheese mat. Previous studies have also reported a strong correlation between the microbes found in ripening cellars and on cheese rinds (13, 58). The impact of the ripening environment on rind microbial communities in the present study was more pronounced for fungi than bacteria and for the cheese rind than the internal sections.

Despite food safety concerns with farmstead cheese production as well as the use of raw milk and wooden tools for cheese production, foodborne bacterial pathogens of concern (i.e., Listeria monocytogenes, *Shigella* spp., Yersinia enterocolitica, Clostridium botulinum, Campylobacter jejuni, Salmonella spp., pathogenic Escherichia coli, coagulase-positive Staphylococcus, and Bacillus cereus) (31) were not identified in any samples in the present study according to the methods and annotation tools described. It is important to emphasize that the sampling was a single point in time and pathogen detection was not an objective of the present study, but this observation is worth noting. Previous work has shown that raw milk from small-scale artisanal cheese farms is typically of high microbiological quality, with total bacteria of <3 log CFU/ml and no detectable foodborne pathogens (31, 59). The absence of foodborne pathogens on wooden tools for cheese production is also consistent with previous studies (2, 12, 21), as biofilms on wooden tool surfaces can function as a dense barrier against pathogen adhesion (56). Biofilm-associated microbes may also provide bioprotection through the production of antimicrobial compounds (60) and the competition for nutrients (61). Thus, it is possible that the overall pathogen prevalence in this farmstead cheese processing continuum, and the related risk of contamination, is low.

### Conclusions.

The results presented here demonstrate the important role of milking environment to the microbial composition of milk. Similarly, they support the bidirectional interaction between the microbial communities in dairy products and cheesemaking tools, whereby contact with milk facilitates growth of certain microbial taxa on the wooden vat. In turn, established communities (e.g., biofilms) on the wooden vat surface inoculate raw milk with technologically important taxa that remain dominant through cheese ripening. This bidirectional interaction also takes place in the ripening cellar with cheese rind microbial communities. The taxa associated with Bethlehem are also similar to those of Saint-Nectaire and Saint-Nectaire-type cheeses without the addition of starter cultures or direct inoculation of any other bacteria or fungi. Altogether, these data highlight the important role of the farmstead cheesemaking environment and traditional processes and tools in the microbial composition of cheese.

## MATERIALS AND METHODS

### Dairy sample collection and preparation.

Sample collection was conducted at the Benedictine Abbey of Regina Laudis in Bethlehem, CT, USA. Dairy product samples (i.e., milk, whey, curd, and cheese) associated with a single cheese production were collected over 2 days to include the evening and morning milk. Samples from the resulting cheeses were collected over time during cheese ripening up to 60 days postmanufacture. All environmental samples, including those in direct and indirect contact with dairy products, were collected on the same day of production. Technical sampling replicates were collected, when possible, as described below and listed in Table S1 in the supplemental material.

Raw milk (∼40 ml) was collected from each teat of four cows (Dutch Belted and Milking Shorthorn) during routine milking by directing the milk stream into sterile 50-ml Falcon centrifuge tubes (Corning, Corning, NY, USA) after initial fore stripping (raw milk, *n* = 4 replicates for sequencing). The remaining milk from each cow was collected in individual stainless steel pails (not sampled), which were then commingled in sets of two by breed in plastic buckets following filtration through a stainless steel milk filter fitted with a paper filter (not sampled). Two samples (∼40 ml each) of filtered milk from each of the two buckets were collected using sterile serological pipettes. Duplicate samples were combined at the time of extraction (filtered milk, *n* = 2). The night prior to cheesemaking, a small portion (∼1 gallon) of the filtered milk was added to the wooden cheese vat and held overnight at ambient temperature, and the remaining portion was stored overnight in a refrigerator for use the following day. Two samples (∼40 ml each) of this milk were collected before being added to the vat. Duplicate samples were combined at the time of extraction (milk before ripening, *n* = 1). Another two samples (∼40 ml each) of this milk were collected the following morning after refrigerated overnight storage and warming in a stockpot to ∼ 40°C. Duplicate samples were combined at the time of extraction (milk stored overnight, *n* = 1). That same morning, two samples (∼40 ml each) of the ripened milk were collected from the wooden vat (ripened milk, *n* = 2). Fresh morning milk (filtered milk) from the day of cheesemaking and the previous evening’s milk that had been refrigerated and then warmed to ∼40°C (milk stored overnight) were added to the ripened milk in the vat (ripened milk) and stirred to combine. Two samples of this mixed milk (∼40 ml each) were collected prior to the addition of coagulant (mixed milk, *n* = 2). The milk was left to coagulate until the desired firmness was reached, at which point the coagulum was broken by hand. Two samples of whey (∼40 ml each) were collected after breaking of the curd, another one was collected during the subsequent step when a wooden paddle (“musador”) was used to stir the curd, and a portion of whey was removed using a metal pan (whey, *n* = 3). Warm water was added around the edges of the curd to keep the environment of the cheese vat warm. Samples of the curd blocks (“tomme”) (cheese curd, ∼50 g) were collected by the cheesemaker using a knife before salting and pressing and placed in sterile Whirl-Pak bags (Nasco, Fort Atkinson, WI, USA) (cheese curd, *n* = 1). All samples were immediately placed on ice, transported directly to the laboratory, and stored at –80°C.

Cheese samples were aseptically collected throughout the ripening process (10 to 12°C and 85 to 90% humidity) on days 0, 4, 7, 14, 21, 44, and 60 using a clean cheese trier sanitized with 70% ethanol. Cheese samples were placed in sterile 50-ml tubes (Corning) to protect their integrity and were immediately stored at –20°C. Cheese samples were aseptically divided at the time of extraction into the following four sections: rind (first ∼3 mm including rind), rind section (∼2 cm from rind sample), middle section (central ∼2 cm), and core (∼2 cm at the end of the sample closest to the center of cheese).

### Environmental sample collection and preparation.

Random grab samples of used bedding (*n* = 2 replicates for sequencing) and feces (*n* = 2) were collected by hand using sanitized gloves and placed in sterile bags. All swab samples were collected with sterile swabs (FLOQswab; Copan Diagnostics, Murrieta, CA, USA) moistened with sterile saline (NaCl at 9 g/liter). One anterior and one posterior teat from each of four cows were sampled individually after routine cleaning by rubbing the swab up and down the entire teat surface from 1 cm above the teat apex to 1 cm below the udder, avoiding contact with the udder hair. Samples from respective teat apices were also collected by rotating a separate swab 360° around the teat canal orifice to a distance of 1 cm above (teat, *n* = 16). Samples from the hands of each milker were collected by swabbing the ventral surface of the hand (including ventral surfaces of the thumb and fingers) after routine washing prior to milking. Swabs from the right and left hands were combined at the time of extraction (milker hands, *n* = 4). Hand samples were also collected from each of the cheesemakers’ left and right hands (ventral surface of the hand, including ventral surfaces of the thumb and fingers) and from the dorsal side of each forearm after routine cleaning before contact with cheese. Swabs from right and left hands and forearms were combined at the time of extraction (cheesemaker hands, *n* = 2). All hand and forearm samples were collected by swabbing in two perpendicular directions and rotating the swab head before changing directions. Floor and wall samples were aseptically collected from two separate 100-cm^2^ areas in each of the three rooms, including the milking barn (floor, *n* = 2; wall, *n* = 2), the cheesemaking room (floor, *n* = 2; wall, *n* = 2), and the ripening cellar (floor, *n* = 2; wall, *n* = 2), as described by Lahou and Uyttendaele (62). Air sampling was conducted based on active impaction of air particles using a filter cassette (37 mm; Sensidyne, St. Peterburg, FL, USA) containing a Teflon filter (0.45-μm pore diameter) for 30 min using a Gil-Air 3 pump (Sensidyne) at 3 liters/min, positioned on a 24-inch tripod (3). The process was repeated in a second location 2 m away. Duplicate samples were combined at extraction (*n* = 1 per location). Air samples were collected in each of the three locations (air milking, *n* = 1; air cheesemaking, *n* =1; air aging, *n* = 1).

Environmental swab samples in the cheesemaking room and ripening cellar were collected following cleaning (if applicable) and before contact with food as previously described (62). A single sample was collected from a 100-cm^2^ section (including floor and wall) of the plastic bucket used to collect filtered milk (bucket; *n* = 1) as well as the metal pan used to remove whey and press curd under the whey (pan, *n* = 1). Given their irregular shapes, samples from the interior and exterior of two plastic cheese hoops and followers used for cheese pressing (hoop, *n* = 2) and the knife blade for cutting the block of curd (knife, *n* = 1) were collected by swabbing an approximately 100-cm^2^ area of the surface in contact with cheese. Samples from the wooden vat were divided into three sections based on visual observation and previous studies (21). Two samples from each of three interior locations in the wooden vat were collected, including the vat floor (vat floor, *n* = 2), lower section of the vat wall (vat bottom, *n* = 2), and top section of the vat wall (vat top, *n* = 2). Samples were also taken from each side of the wooden paddle (100-cm^2^ area each) used to stir and collect curds (paddle, *n* = 2). Samples from wooden surfaces, including the vat and paddle, were collected by first brushing the designated area with a sterile brush moistened with sterile saline to release microbes within the wood followed by swabbing (21). Two samples of tap water (∼40 ml each) from the cheesemaking room that was used for cleaning and added to the vat to maintain the temperature of the curd mass were collected and combined at extraction (water, *n* = 1). Salt (∼50 g, *n* = 1) used for salting pressed cheese and a single tablet of animal rennet (coagulant, *n* = 1) (Walcoren, Quebec City, Quebec, CA) were aseptically placed in Whirl-Pak bags. One sample from the wooden ripening shelf (100-cm^2^ area) was collected before the addition of the bamboo mat with cheese (shelf, *n* = 1) using the previously described swabbing technique. One sample from the bamboo cheese mat directly in contact with cheese during ripening was collected as described for wooden tools. Shards from the mat were also collected with a sterile razor blade (cheese mat, *n* = 2).

### Genomic DNA extraction and bacterial 16S rRNA V4 and fungal ITS2 amplification and sequencing.

Genomic DNA was extracted from replicate samples or pooled replicates (Table S1, sampling replicates and sequencing replicates) using a DNeasy PowerFood microbial kit (Qiagen, Hilden, Germany). Extracted DNA samples were amplified and sequenced at the University of Connecticut Microbial Analysis, Resources, and Services Center using the standard protocol. DNA was quantified using Pico-Green fluorescent dye (Invitrogen, Waltham, MA, USA) and was used as the template to amplify the bacterial 16S rRNA V4 region (515F and 806R) and fungal internal transcribed spacer regions (ITS3 and ITS4). Primers with Illumina adapters and dual barcodes were used for amplification. PCR conditions for bacteria consisted of 94°C for 2 min, 30 cycles of 30 s at 94°C, 30 s at 53°C, and 60 s at 72°C, and a final extension at 72°C for 5 min. PCR conditions for fungi consisted of 95°C for 2 min, 5 cycles of 30 s at 95°C, 60 s at 48°C, and 60 s at 72°C, 25 cycles of 30 s at 95°C, 60 s at 55°C, and 60 s at 72°C, and a final extension at 72°C for 5 min. PCR products were cleaned using Mag-Bind RxnPure plus (Omega Bio-tek, Norcross, GA, USA) and sequenced on Illumina MiSeq using the 2 × 250-bp kit (Illumina, Inc., San Diego, CA, USA). Negative extraction controls were also sequenced to test for contamination.

### Data processing and analyses.

Raw sequences were preprocessed using the DADA2 package (v. 1.9.0) (63) in R (v. 3.4.3) (64). Bacterial forward and reverse sequences were trimmed at 240 bp and 160 bp based on quality profiles of sequences. Fungal sequences were untrimmed. Sequences with ambiguous bases and more than two expected errors were removed. The DADA2 core sample inference algorithm was applied with pseudopooling of information across samples. Denoised forward and reverse sequences were merged and chimeras were removed. Bacterial taxonomy was assigned based on the Silva database (v. 132) (65) and the dairy database, DAIRYdb (66). Amplicon sequence variants (ASVs) aligning to archaea, eukaryotes, chloroplasts, and mitochondria were removed. Fungal taxonomy referred to the UNITE database (v. 8.0) (67). Only ASVs aligned to the Fungi kingdom were retained for analysis.

Diversity analysis was conducted in R using the Phyloseq package (v. 1.25.2) (68) and visualized using the ggplot2 package (v. 3.4.4) (69). The data set was not rarefied, as it could result in a high rate of false positives and omit samples from accurate clustering (70). Alpha diversity was measured by the Shannon index, which indicates richness and evenness and is less dependent on the number of sequences per sample. Analysis of variance or Kruskal-Wallis analysis was conducted to determine if alpha diversity of environmental samples differed between processing rooms. Beta diversity was measured as the Bray-Curtis, Jaccard, weighted UniFrac, and unweighted UniFrac distances. Ordination was performed on the distance matrices and visualized by principal coordinate analysis (PCoA) plots.

Pairwise permutational multivariate analysis of variance (PERMANOVA; 999 permutations) was performed on distance matrices using the pairwiseAdonis package (v 0.0.1) to statistically test microbial structure differences between processing rooms (71). To identify taxa contributing to the variation in community composition between processing rooms, pairwise comparisons of ASV abundance were conducted on environmental samples between milking barn-cheesemaking room, cheesemaking room-ripening cellar, and milking barn-ripening cellar. Two differential abundance analyses were used in the study. A conventional tool, DESeq2 package (v. 1.18.1) (72), was conducted on the unrarefied data set, and ASVs were filtered using an adjusted *P *value of <0.01 and a log_2_ fold change greater than 2. A compositional method, ALDEx2 package (v. 1.10.0) (73), was also carried out. Instead of modeling the data set as count number, it models the data as log ratio transformed probability distribution for analysis (74). The data set was first centered and log ratio transformed by 128 Monte Carlo replicates and Welch’s *t* tests, the Wilcoxon rank tests were performed, and effect size difference of  ≥1 was reported. Distance tree on different sections of cheeses over time were constructed by unweighted pair group method using average linkages (UPGMA).

SourceTracker, a Bayesian-based algorithm (55), was used to identify microbial dispersal from house sources (i.e., processing environment) and estimate their contribution to the microbial populations in dairy products. Analyses were conducted at each step along the cheesemaking continuum, with each dairy sample considered a sink in the following order: raw milk, unripened milk (filtered milk, milk before ripening, milk stored overnight), ripened milk, mixed milk, cheese curd, and cheese on day 60. The preceding dairy sample and all environmental samples in direct contact with the dairy sample were considered possible sources of the subsequent dairy samples.

### Ethics statement.

All protocols involving human subjects were approved by the Institutional Review Board of The University of Connecticut (protocol H2O-0161). Verbal and signed informed consent was obtained from all subjects.

### Data availability.

Raw sequence data, except for those associated with samples from human subjects, are available at NCBI (SRA accession no. PRJNA759702). All scripts for analyses were deposited on Github at https://github.com/langsun94/Bethlehem.
