# Supplementary material for: Composition, Succession, and Source Tracking of Microbial Communities throughout the Traditional Production of a Farmstead Cheese
Source: mSystems. 2021 Sep 28;6(5):e00830-21. doi: 10.1128/mSystems.00830-21 (PMC8547439; doi:10.1128/mSystems.00830-21)
Supplement: TABLE S1 [file msystems.00830-21-st001.docx]

Table S1.

| **Sampling ID** | **Processing location** | **Sampling replicates** | **Sequencing replicates** | **Successfully sequenced for bacteria v4** | **Successfully sequenced for fungal ITS** |
| --- | --- | --- | --- | --- | --- |
| rawmilk | Milking_barn | 4 | 4 | 2 | 1 |
| filtered_milk | Milking_barn | 4 | 2 | 2 | 2 |
| milk_before_ripening | Milking_barn | 2 | 1 | 1 | 1 |
| milk_stored_overnight | Milking_barn | 2 | 1 | 1 | 0 |
| ripened_milk | Cheesemaking | 2 | 2 | 2 | 2 |
| mixed_milk | Cheesemaking | 2 | 2 | 2 | 2 |
| whey | Cheesemaking | 3 | 3 | 3 | 2 |
| cheese_curd | Cheesemaking | 1 | 1 | 1 | 1 |
| cheese_day0_core | Ripening_cellar | 1 | 1 | 1 | 0 |
| cheese_day0_middlesection | Ripening_cellar | 1 | 1 | 1 | 1 |
| cheese_day0_rind | Ripening_cellar | 1 | 1 | 1 | 1 |
| cheese_day0_rindsection | Ripening_cellar | 1 | 1 | 1 | 1 |
| cheese_day4_core | Ripening_cellar | 1 | 1 | 1 | 1 |
| cheese_day4_middlesection | Ripening_cellar | 1 | 1 | 1 | 1 |
| cheese_day4_rind | Ripening_cellar | 1 | 1 | 1 | 1 |
| cheese_day4_rindsection | Ripening_cellar | 1 | 1 | 1 | 1 |
| cheese_day7_core | Ripening_cellar | 1 | 1 | 1 | 1 |
| cheese_day7_middlesection | Ripening_cellar | 1 | 1 | 1 | 1 |
| cheese_day7_rind | Ripening_cellar | 1 | 1 | 1 | 1 |
| cheese_day7_rindsection | Ripening_cellar | 1 | 1 | 1 | 1 |
| cheese_day14_core | Ripening_cellar | 1 | 1 | 1 | 1 |
| cheese_day14_middlesection | Ripening_cellar | 1 | 1 | 1 | 1 |
| cheese_day14_rind | Ripening_cellar | 1 | 1 | 1 | 1 |
| cheese_day14_rindsection | Ripening_cellar | 1 | 1 | 1 | 1 |
| cheese_day21_core | Ripening_cellar | 1 | 1 | 1 | 1 |
| cheese_day21_middlesection | Ripening_cellar | 1 | 1 | 1 | 0 |
| cheese_day21_rind | Ripening_cellar | 1 | 1 | 1 | 1 |
| cheese_day21_rindsection | Ripening_cellar | 1 | 1 | 1 | 1 |
| cheese_day44_core | Ripening_cellar | 1 | 1 | 1 | 1 |
| cheese_day44_middlesection | Ripening_cellar | 1 | 1 | 1 | 1 |
| cheese_day44_rind | Ripening_cellar | 1 | 1 | 1 | 1 |
| cheese_day44_rindsection | Ripening_cellar | 1 | 1 | 1 | 1 |
| cheese_day60_core | Ripening_cellar | 1 | 1 | 1 | 1 |
| cheese_day60_middlesection | Ripening_cellar | 1 | 1 | 1 | 1 |
| cheese_day60_rindsection | Ripening_cellar | 1 | 1 | 1 | 1 |
| cheese_day60_rind | Ripening_cellar | 1 | 1 | 1 | 1 |
| air_milking | Milking_barn | 2 | 1 | 1 | 1 |
| floor_milking | Milking_barn | 2 | 2 | 2 | 2 |
| wall_milking | Milking_barn | 2 | 2 | 2 | 2 |
| bucket | Milking_barn | 1 | 1 | 1 | 1 |
| milker_hands | Milking_barn | 8 | 4 | 3 | 3 |
| teat | Milking_barn | 16 | 16 | 10 | 12 |
| bedding | Milking_barn | 2 | 2 | 2 | 2 |
| feces | Milking_barn | 2 | 2 | 2 | 2 |
| air_cheesemaking | Cheesemaking | 2 | 1 | 1 | 1 |
| floor_cheesemaking | Cheesemaking | 2 | 2 | 2 | 2 |
| wall_cheesemaking | Cheesemaking | 2 | 2 | 2 | 1 |
| cheesemaker_hands | Cheesemaking | 4 | 2 | 2 | 2 |
| coagulant | Cheesemaking | 1 | 1 | 1 | 0 |
| hoop | Cheesemaking | 2 | 2 | 2 | 1 |
| knife | Cheesemaking | 1 | 1 | 1 | 1 |
| paddle | Cheesemaking | 2 | 2 | 2 | 2 |
| pan | Cheesemaking | 1 | 1 | 1 | 1 |
| salt | Cheesemaking | 1 | 1 | 0 | 0 |
| water | Cheesemaking | 2 | 1 | 1 | 1 |
| vat_bottom | Cheesemaking | 2 | 2 | 2 | 2 |
| vat_floor | Cheesemaking | 2 | 2 | 2 | 2 |
| vat_top | Cheesemaking | 2 | 2 | 2 | 2 |
| air_aging | Ripening_cellar | 2 | 1 | 1 | 1 |
| floor_aging | Ripening_cellar | 2 | 2 | 2 | 2 |
| wall_aging | Ripening_cellar | 2 | 2 | 2 | 2 |
| cheesemat | Ripening_cellar | 2 | 2 | 2 | 2 |
| shelf | Ripening_cellar | 1 | 1 | 1 | 1 |
| **Total nubmer** |  | 118 | 104 | 94 | 88 |
