# Supplementary material for: Composition, Succession, and Source Tracking of Microbial Communities throughout the Traditional Production of a Farmstead Cheese
Source: mSystems. 2021 Sep 28;6(5):e00830-21. doi: 10.1128/mSystems.00830-21 (PMC8547439; doi:10.1128/mSystems.00830-21)
Supplement: TABLE S2 [file msystems.00830-21-st002.docx]

Table S2.

|  | Bray–Curtis | | Jaccard | | Unifrac weighted | | Unifrac unweighted | |
| --- | --- | --- | --- | --- | --- | --- | --- | --- |
| Environmental samples | R2 | *p* (adjusted) | R2 | *p* (adjusted) | R2 | *p* (adjusted) | R2 | *p* (adjusted) |
| Milking_barn vs. Cheesemaking | 0.124 | 0.003 | 0.122 | 0.003 | 0.122 | 0.003 | 0.122 | 0.003 |
| Milking_barn vs. Ripening_cellar | 0.105 | 0.003 | 0.105 | 0.003 | 0.105 | 0.003 | 0.105 | 0.003 |
| Cheesemiking vs. Ripening_cellar | 0.113 | 0.006 | 0.113 | 0.003 | 0.113 | 0.003 | 0.113 | 0.006 |
